# A Systems Biology Strategy for Predicting Similarities and Differences of Drug Effects: Evidence for Drug-specific Modulation of Inflammation in Atherosclerosis

**DOI:** 10.1186/1752-0509-5-125

**Published:** 2011-08-12

**Authors:** Robert Kleemann, Svetlana Bureeva, Ally Perlina, Jim Kaput, Lars Verschuren, Peter Y Wielinga, Eva Hurt-Camejo, Yuri Nikolsky, Ben van Ommen, Teake Kooistra

**Affiliations:** 1 Metabolic Health Research, TNO, Zernikedreef 9, Leiden, 2333 CK, The Netherlands; 2 Microbiology and Systems Biology, TNO, Utrechtseweg 48, Zeist, 3704 HE, The Netherlands; 3 GeneGo, Inc., 5901 Priestly Drive, Carlsbad, CA 92008, USA; 4 Division of Personalized Nutrition and Medicine FDA, National Center for Toxicological Research, 3900 NCTR Road, Jefferson, AR 72079, USA; 5 Nestle Institute of Health Sciences, EPFL Campus Bâtiment G at Quartier de l'Innovation, Lausanne, 1015, Switzerland; 6 Cardiovascular & Gastrointestinal Innovative Medicines Unit, AstraZeneca, Pepparedsleden 1, Mölndal, 43183, Sweden

## Abstract

**Background:**

Successful drug development has been hampered by a limited understanding of how to translate laboratory-based biological discoveries into safe and effective medicines. We have developed a generic method for predicting the effects of drugs on biological processes. Information derived from the chemical structure and experimental omics data from short-term efficacy studies are combined to predict the possible protein targets and cellular pathways affected by drugs.

**Results:**

Validation of the method with anti-atherosclerotic compounds (fenofibrate, rosuvastatin, LXR activator T0901317) demonstrated a great conformity between the computationally predicted effects and the wet-lab biochemical effects. Comparative genome-wide pathway mapping revealed that the biological drug effects were realized largely via *different *pathways and mechanisms. In line with the predictions, the drugs showed differential effects on inflammatory pathways (downstream of PDGF, VEGF, IFNγ, TGFβ, IL1β, TNFα, LPS), transcriptional regulators (NFκB, C/EBP, STAT3, AP-1) and enzymes (PKCδ, AKT, PLA2), and they quenched different aspects of the inflammatory signaling cascade. Fenofibrate, the compound predicted to be most efficacious in inhibiting early processes of atherosclerosis, had the strongest effect on early lesion development.

**Conclusion:**

Our approach provides mechanistic rationales for the differential and common effects of drugs and may help to better understand the origins of drug actions and the design of combination therapies.

## Background

In addition to their established pharmacological activities, many preclinical and commercial drugs exert effects that are not predictable from their presumed mode of action and primary target [1-3]. Unanticipated effects represent both opportunities and challenges for modern drug development and for health outcomes. Off-target effects can lead to new therapeutic applications and repositioning of existing drugs [1]. However, unexpected effects can also be responsible for adverse drug events, low patient compliance and, in case of severe side effects, withdrawal from clinical testing or the market [4,5].

The molecular causes for the positive as well as negative off-target effects are largely unexplored. Obviously, metabolic transformations of pharmaceuticals can profoundly impact their bioavailability, efficacy and chronic toxicity, and both the parent molecule and the products of metabolic transformations can interfere with endogenous metabolism [6]. More recent protein-ligand interaction studies provide another molecular rationale for unanticipated drug effects showing that small molecule drugs can bind protein targets which lack obvious sequence or structural similarity and which are involved in entirely different pharmacology [7]. Thus, drugs and their metabolites can be active on multiple direct and indirect targets involved in many dozens of pathways, which makes it crucial to be able to understand or predict the on- and off-target effects of a particular drug.

Here we present a systems biology-based strategy that allows prediction of shared and differential effects of drugs. The approach uses information derived from the chemical structure of the drugs together with experimental omics data from short-term intervention studies. Because of the general relevance and global burden of cardiovascular disease [8], the present study was performed in a setting of experimental atherosclerosis using an established disease model, ApoE3Leiden mice which exhibit a unique human-like sensitivity to cardiovascular drugs [9]. Three prototype cardiovascular drugs were tested: a statin (rosuvastatin; RSV), a fibrate (fenofibrate; FF) and a liver-X-receptor (LXR)-agonist (T0901317;N-(2,2,2-Trifluoro-ethyl)-N-[4-(2,2,2-trifluoro-1-hydroxy-1-trifluoromethyl-ethyl)-phenyl]-benzene-sulfonamide; T09). The liver is the primary target organ for all three drugs, but they differ in their mechanism of action, i.e. how they alter hepatic lipid metabolism and how they attenuate atherosclerosis [10-12]. At the doses employed, the drugs can exert anti-inflammatory activities [13-15] that may contribute to their anti-atherosclerotic effect.

On-target and off-target effects of the three drugs were predicted through the similarity of their chemical structure (chemical similarity search of parent compounds and their metabolites against a MetaDrug™ database[16]) and their induced hepatic transcriptome profile. The *predicted *biological effects were then compared with *real *experimental outcomes in ApoE3Leiden mice, viz. plasma lipid levels, inflammation marker concentrations, transcription factor activities, and aortic atherosclerotic lesions. With respect to delineating the similarities and differences between the three drugs, particular emphasis was put on inflammatory aspects, because their precise anti-inflammatory action is not fully understood. For example, it was unclear whether the three drugs impact on similar or complementary inflammatory pathways.

This study provides evidence for a concordance of predicted activities and experimental biochemical effects thereby exemplifying the power of computational strategies for efficacy prediction and the role that systems biology may have in future drug discovery.

## Results

### Chemical structure-based and transcriptome-based prediction of genes affected by drugs

**Figure 1:**
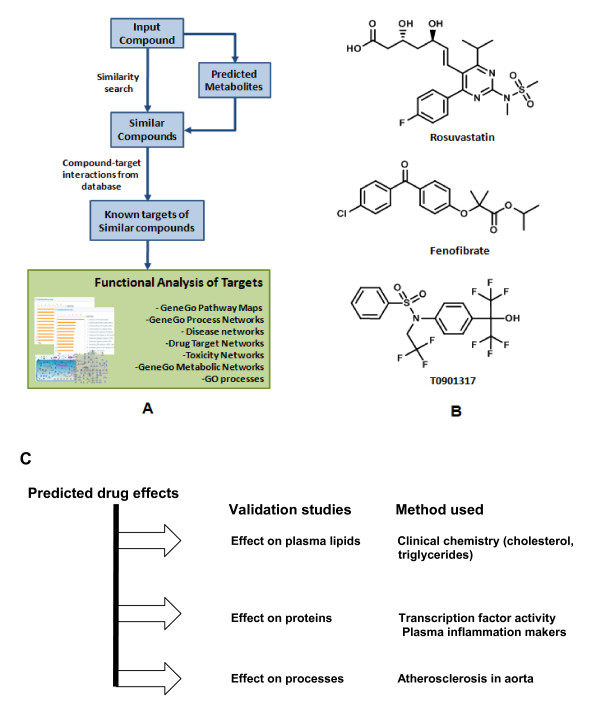
**Workflow for prediction of biological effects of drugs**. **A**, The workflow prediction of biological effects based on an arbitrary chemical structure. **B**, Molecular structures of rosuvastatin (RSV), fenofibrate (FF), LXR-activator T0901317 (T09). The structures were uploaded into MetaDrug™ and a similarity search was performed based on the structures of the input compounds (with the Tanimoto coefficient, used to calculate similarities and differences, set at 0.7). The direct and indirect targets provided were used for enrichment analysis. **C**, Overview of the biochemical analyses that were performed to validate the predictions on different levels (plasma lipids, protein activity and expression, endpoint disease).

Figure 1 provides a conceptual overview of the *in silico *analyses to predict drug effects based on their chemical structure and it illustrates the subsequent biochemical validation experiments on the level plasma markers, liver proteins and cardiovascular endpoints.

**Figure 2:**
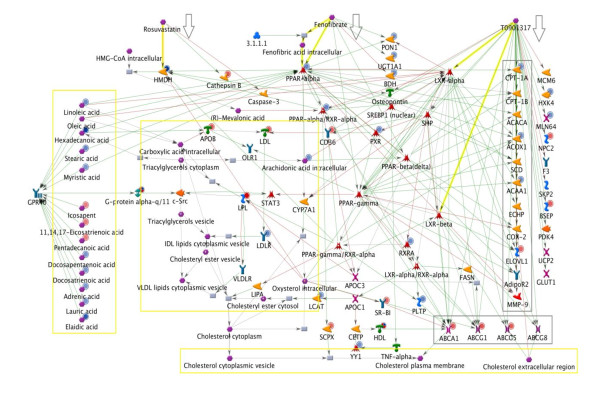
**Main direct and indirect targets of the drugs**. Network representing the main direct and indirect targets of rosuvastatin (RSV), fenofibrate (FF) and T0901317 (T09) with overlaid high-cholesterol expression and metabolomic data from ArrayExpress database with accession number: ETABM-253 [27]. Drugs and metabolites are depicted in purple (hexagonal symbols), factors with catalytic or enzymatic activity in yellow, transporters in purple (cross symbols), transcription factors in red (flash star symbol), cytokines and lipoproteins in green, receptors and adaptor proteins in blue. Red (blue) circles in the top-right corner of each icon indicate a positive (negative) effect of high-cholesterol on the expression level. Green (red) lines indicate an activating (inhibiting) effect between interacting molecules. Bold yellow arrows specify the primary drug targets (HMDH for RSV, PPAR-alpha for FF, LXR-alpha/LXR-beta for T0901317). Lipids and molecules involved in basic lipoprotein metabolism are boxed in yellow. Grey boxes represent shared targets, i.e. factors that are affected by at least two of the drugs. Factors that are regulated specifically by T0901317 are arranged vertically on the right (not boxed).

To visualize the known molecular mechanisms of action of RSV, FF and T09 we built a network summarizing their direct and remote targets as well as their interactome neighborhood (Figure 2).

RSV is a structural inhibitor of 3-hydroxy-3-methylglutaryl coenzyme A reductase (HMDH), the rate-limiting enzyme for hepatic cholesterol biosynthesis. Inhibition of this enzyme by RSV results in decreased cholesterol biosynthesis and upregulation of the LDL receptor, and consequently predicts a reduction of plasma cholesterol. The indirect effects of RSV effects are predicted or known to occur through Caspase 3, Cathepsin B [17] and other proteins (not shown).

Fenofibrate (FF) is converted in liver into its active metabolite, fenofibric acid. Fenofibric acid is a ligand and an agonist of the lipid sensor receptor PPARα which reduces the expression of apolipoprotein C-III (APOC3). Fenofibric acid can also activate PPARβ/δ and PPARγ but with lower affinity (>10-fold lower than for PPARα). FF indirectly modulates the activity or expression level of several other genes, including 3-hydroxybutyrate dehydrogenase[18], paraoxonase-1, a serum high-density lipoprotein-associated phosphotriesterase[19], as well as several cytokines and chemokines [20] (not shown). FF increases the catabolism of triglycerides by induction of lipoprotein lipase (LPL) and reduces the production of triglyceride-rich lipoproteins (VLDL). The predicted net effect of FF on circulating lipids is a specific reduction of plasma triglycerides and cholesterol.

T09 activates liver × receptor (LXR) alpha/beta nuclear receptors, which are intracellular sterol sensors that regulate expression of genes controlling cholesterol and bile metabolism. T09 influences expression of a number of organic transporters, including those reported to participate in lipid transport (ABCG1 [21], ABCG5, ABCG8 [22], ABCA1), enzymes involved in lipid metabolism (ACOX-1, ECHP, ACAA1 [23]), and inflammatory molecules (COX-2, MMP-9 [24]). T09 also antagonizes pregnane × receptor [25], an important component of the adaptive defense mechanism against toxic substances. Through co-ordination of the expression of target genes in multiple tissues (not shown), T09 is predicted to increase cholesterol efflux from the peripheral organs into the circulation. Since the hepatic clearance receptors for LDL-cholesterol are not affected by T09, the predicted net effect is an elevation of plasma cholesterol.

To validate the predicted effects, we analyzed plasma lipids of ApoE3Leiden mice treated with an atherogenic high cholesterol diet in the presence or absence of RSV, FF and T09. Data of plasma cholesterol and plasma triglycerides are provided in Additional file 1 (Table S1). In accord with the prediction, RSV significantly reduced plasma cholesterol levels. FF-treated E3L mice displayed significantly lower triglyceride and cholesterol levels. By contrast, T09 treatment increased plasma cholesterol levels and also increased plasma triglycerides.

### Prediction of compound similarities on the level of biological processes and pathways

In order to predict the similarities and differences of RSV, FF and T09 action, we applied ontology enrichment analysis to two kinds of gene lists: (i) lists of putative targets predicted from the chemical structure and (ii) lists of potential targets obtained from the hepatic gene expression profile.

The chemical structure generated lists of putative targets for each drug (Additional file 2) were generated by searching their chemical structures for similar compounds with known targets in the MetaDrug database (Figure 1; details in Methods).

The expression-generated lists represented differentially expressed genes (DEGs) in mouse liver after 10 weeks of treatment with cardiovascular drug-containing diet, measured by microarrays (Additional file 2). The two lists were generated independently using different techniques and data sources. Therefore, any concordance in enrichment distributions on pathways, normal and toxicity processes, or disease biomarker ontology (as examples), is independent evidence for not only identifying a specific ontological entity but also for explaining both on-target and off-target effects.

**Figure 3:**
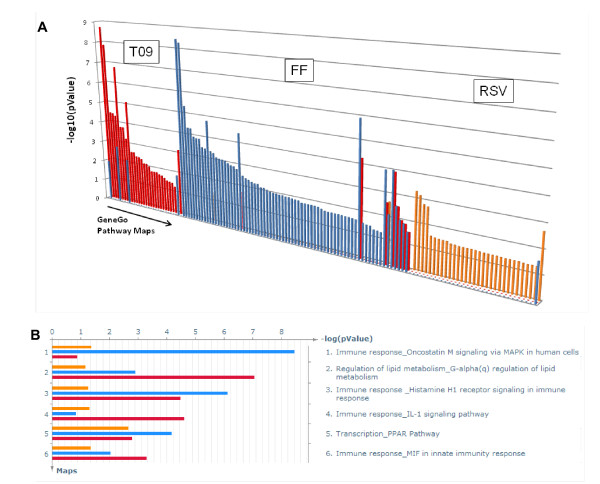
**Predicted biological target processes and pathways**. Overview of the predicted biological target processes and pathways obtained from enrichment analysis. Information derived from the chemical structure of the drugs was used to predict possible targets. These possible targets were then used to predict target processes and pathways for rosuvastatin (RSV; orange), fenofibrate (FF; blue) and LXR activator T0901317 (T09; red). **A: **Target distribution overview demonstrating that each of the drugs has a characteristic target pattern with little overlap between the drugs; **B: **Most significant common pathway maps for RSV, FF and T09.

Next, the lists of known targets for the three compounds were computationally expanded to 'possible targets' defined as targets of compounds similar to RSV, FF and T09. As most compounds similar to RSV belonged to the class of statins and most compounds similar to FF to the class of fibrates, this expansion allows generalization of the analysis and comparison of compound classes (e.g. statins vs. fibrates). The largest number of possible direct and indirect targets was identified for FF, including extracellular signal-regulated kinases 1 and 2, elastases and lipoprotein lipase (not shown). All possible direct/indirect targets of the drugs (not shown) were subjected to enrichment analysis and mapped to biological processes for visualization (Figure 3A). The length of the colored bars (orange - RSV, blue - FF, red - T09) corresponds to the number of targets in a process map. Importantly, only a few maps were common to the three compounds and most pathways were affected by one specific drug. This indicates that biological effects are realized via *different *pathways and mechanisms and also implies that putative and unexpected off-target effects of the three drugs are likely to be different.

The few common maps included '*Lipid metabolism*' and '*Immune response*' which correlates well with the reported pharmacological compound action and the well-documented correlation between lipid metabolism and inflammation [26,27]. Among the most significant processes predicted to be affected by all three drugs are interleukin-1 signaling, oncostatin M signaling, histamin H1 receptor signaling and macrophage migration inhibitory factor (MIF) signaling as well as transcriptional regulation through peroxisome proliferator-activated receptors (PPARs) (Figure 3B).

### Prediction of drug-specific effects on the level of biological processes and pathways

Prediction analysis showed that when compared with RSV and T09, FF affected the most biological pathways and processes (Figure 3A). To refine this prediction and identify which pathways and processes are specifically affected by FF and not by the other two drugs, the list of FF-modulated targets was computationally expanded by their first-step physical protein-protein interactions with their network neighbors. Similar expansions were performed for RSV and T09.

**Figure 4:**
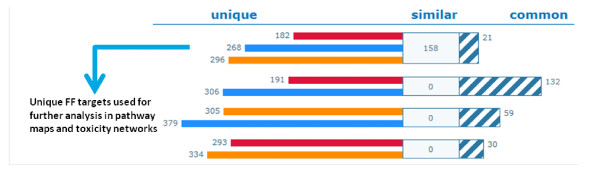
**Distribution and numbers of unique, similar and common targets**. Target intersection results: common targets - striped bar, similar targets - white bar, unique targets - rosuvastatin (RSV; orange), fenofibrate (FF; blue) and LXR activator T0901317 (T09; red). Blue arrow shows that the 268 targets unique for FF were selected for a subsequent more detailed enrichment analysis focusing on pathways and toxicity. Common - genes that are common between all three compounds. Similar - genes that are common between any of compound pairs.

The expansions resulted in three types of possible targets: (i) shared by all three drugs; (ii) shared by two drugs (i.e. by the pairs FF and T09; RSV and T09; FF and RSV) and (iii) unique for a drug (Figure 4; individual target lists not shown). Based on this prediction, only 21 common targets were identified, among which are PPARγ, CCAAT-enhancer-binding proteins (C/EBP), SMAD3, tumor suppressor p53, SP1, androgen receptor, retinoblastoma protein, cyclooxygenase-2, ERK1/2 and Jun N-terminal kinase.

To identify the pathways and processes predominantly affected by FF, we performed enrichment analysis based on the unique targets for FF (268 proteins; blue arrow in Figure 4). The canonical pathway maps and the toxicity network ontologies indicated inflammation-related processes (e.g., cell adhesion and IL-6 signaling) among the most significant processes affected by FF, and not by the other compounds (Table 1). Thus, based on this prediction, one would expect that FF differs in its impact on inflammation when compared to the other two drugs.

**Table 1:** Prediction of the most significant pathways and networks unique for fenofibrate.

GeneGO Pathway Maps	pValue	GeneGo Toxicity Networks	pValue
Development: Growth hormone signaling via PI3K/AKT and MAPK cascades	1.152e-10	Blood coagulation: Coagulation factors. Plasminogen signaling	1.696e-08
Development: IGF-RI signaling	1.757e-08	Signal transduction: Janus kinase 2 (protein tyrosine kinase)	6.223e-08
Cytokine production by Th17 cells in CF	2.043e-08	Proliferation: Lymphocyte proliferation_STATs	2.808e-07
Cell adhesion: ECM remodeling	2.142e-08	Inflammation: SOCS3 in JAK-STAT cascade	4.345e-07
Cell adhesion: Chemokines and adhesion	2.233e-08	Inflammation: Kallikreins signaling	4.432e-07
Immune response : IL-4 - antiapoptotic action	3.753e-08	Inflammation: SERPINA3 regulation	6.102e-07
Transcription: Androgen Receptor nuclear signaling	7.793e-08	Signal transduction: IL-6R signaling ; hemopexin	9.601e-07
Transcription: Receptor-mediated HIF regulation	3.440e-07	Signal transduction: IL-6R signaling; APCS	9.601e-07
Cell adhesion: PLAU signaling	3.440e-07	Signal transduction : IL-6R signaling ; haptoglobin	1.478e-06
Immune response: IL-6 signaling pathway	5.856e-07	Proliferation: Positive regulation, HGF, CRIPTO, CCL14, IP10 signaling	2.302e-06

Unique fenofibrate targets (from prediction analysis) were employed to GeneGo Pathway Maps and GeneGo Toxicity Networks. The 10 most significant pathways maps and toxicity networks are shown.

### Validation of the predicted similarities and differences of drug effects

To validate the predictions made above, we plotted the liver microarray data of the three drugs on key networks and pathways identified previously [27], particularly emphasizing commonalities and differences related to inflammation. The gene expression analysis was paralleled by a comparison of biochemical parameters, i.e. plasma inflammation markers (by ELISA) and transcription factor activity in liver homogenates (by TransAM).

**Figure 5:**
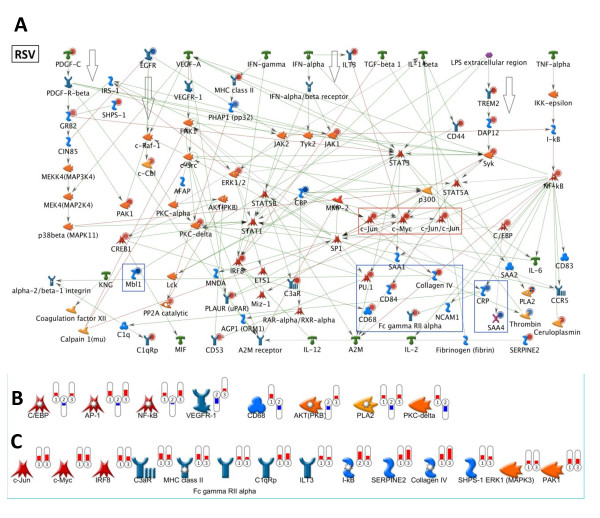
**Effect of rosuvastatin on inflammatory networks and pathways**. Major hepatic inflammatory pathways activated by high cholesterol (HC) feeding (identified in [27]) were plotted in one graph and the effects of cardiovascular drugs were analyzed. White arrows indicate the pathways that were active in presence of a drug. Cytokines and chemokines are shown in green, transcription factors in red, adaptor molecules in blue, factors with catalytic or enzymatic activity in yellow. Filled red circles (blue circles) indicate that expression of a factor is upregulated (downregulated). Red (blue) boxes indicate upregulated (downregulated) gene clusters. **A**, Pathways and transcription factors affected by rosuvastatin (RSV). **B**, Genes from the network regulated differentially by RSV, FF and T09 with thermometers 1,2 and 3 indicating RSV, FF, T09, respectively. Red (blue) color of a thermometer indicates upregulation (downregulation). **C**, Genes upregulated only by RSV and T09.

**Figure 6:**
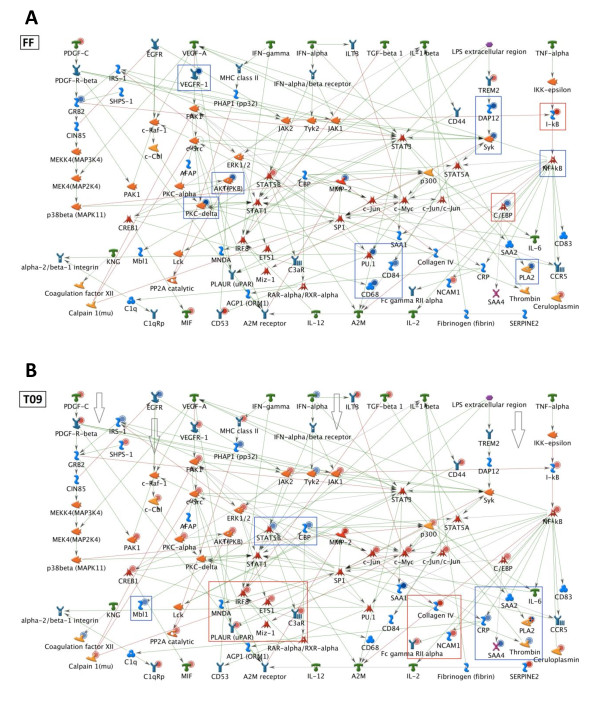
**Effect of fenofibrate and LXR agonist T0901317 on inflammatory networks and pathways**. Pathways and transcription factors modulated by **A**, fenofibrate (FF) and **B**, LXR agonist T0901317 (T09) under HC conditions essentially as described in Figure 5.

In line with the predicted similarities, all three drugs affected inflammatory processes controlled by IL-1 and MIF and mediated by C/EBP, SP1, ERK1/2 and JNK. Also in agreement with predictions, each drug acted in a selective fashion and quenched different regions of the inflammatory network (blue boxes in Figures 5 and 6). A more granular analysis focusing on single genes in networks revealed that RSV and T09 also exhibited similarities while FF frequently showed opposite effects. For example, RSV and T09 enhanced gene expression of the inflammatory transcription factors NFκB, c-Jun and C/EBP while FF did not (Figures 5 and 6). FF also quenched the STAT3 signaling pathway while that pathway remained active for RSV and T09.

**Figure 7:**
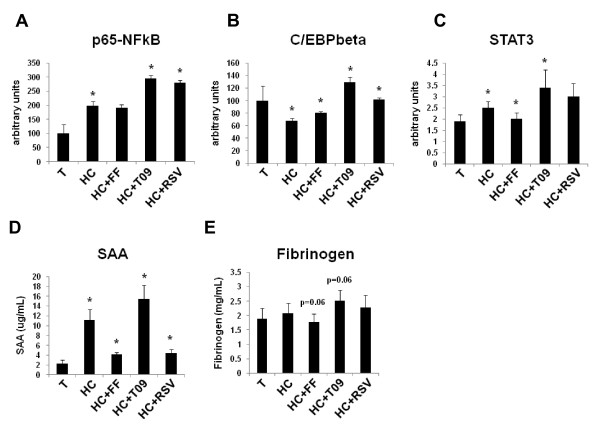
**Biochemical validation of differential effects of RSV, FF and T09 on inflammation**. Quantification of transcription factor activity in liver homogenates and analysis of liver-derived inflammation markers in plasma. ApoE3Leiden mice treated with chow control diet T (for baseline levels) or an atherogenic high cholesterol diet (HC) in the presence or absence of rosuvastatin (RSV); fenofibrate (FF) and LXR agonist T0901317 (T09). **A**, p65-NFκB; **B**, C/EBPβ; **C**, p-STAT3 and circulating markers **D**, SAA; **E**, fibrinogen.

Biochemical quantification of transcription factor activity in liver homogenates confirmed these findings: RSV and T09-treated livers showed greater transcriptional activity for p65-NFκB, C/EBPβ and STAT3 than FF (Figure 7A-C). Specifically for FF, the pathways leading to C/EBP and STAT3 and relevant for IL-6 signaling were not activated and the inhibitor of nuclear factor kappa B, IκBα, was up-regulated at the level of mRNA abundance (Figure 6A). Although STAT3 activity is predicted by the network to be quenched, the transcriptional activity of STAT3 was not significantly reduced compared to HC. Direct measures of active, phosphorylated IκBa protein in liver homogenates of FF-treated mice showed an increase (~20%), confirming changes in gene expression (not shown).

The pathway maps and biochemical data both demonstrated the same differential effect of RSV, FF and T09 on inflammatory pathways: RSV and T09 mainly suppressed acute inflammatory effects while FF suppressed both acute and chronic inflammatory processes. Indeed, the plasma levels of the chronic inflammation markers SAA and fibrinogen, an IL-6-dependent acute phase protein, were lower with FF (SAA; P<0.05 vs HC and fibrinogen; P = 0.06 vs HC) but not with T09 (Figure 7D/E). RSV lowered SAA but also not fibrinogen.

### FF exhibits reciprocal expression patterns in comparison to RSV and T09

**Figure 8:**
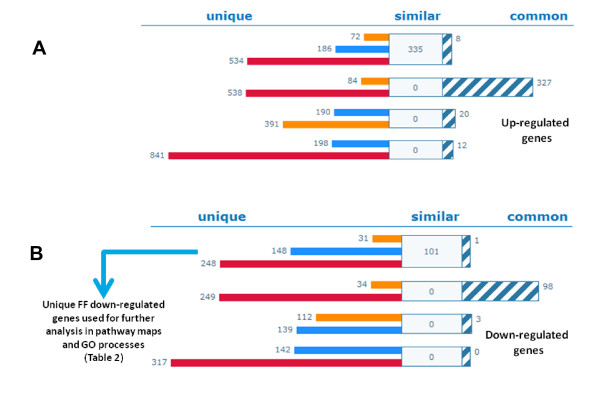
**Validation of differentially expressed genes**. Analyses of genes differentially expressed (up- or down-regulated) with a threshold of 2.1-fold (1.1 Log2ratio). Target intersection results: common targets - striped bar, unique targets - rosuvastatin (RSV, orange), fenofibrate (FF, blue), LXR agonist T0901317 (T09, red); **A: **up-regulated genes; **B: **down-regulated genes. Common - genes that are common between all three compounds. Similar - genes that are common between any of compound pairs.

Analysis of *all *differentially expressed genes (DEGs) from microarray analysis showed most similarities between RSV and T09 (Figure 8A upregulated genes and 8B downregulated genes). RSV and T09 change expression of the same genes in the same direction with 327 commonly upregulated and 98 commonly downregulated. The FF gene expression profile is entirely different and FF suppressed many of the genes that are activated by RSV and T09. This general finding is consistent with the similarities between RSV and T09 on certain inflammatory networks (Figure 5 and 6) and the different effect of FF.

**Figure 9:**
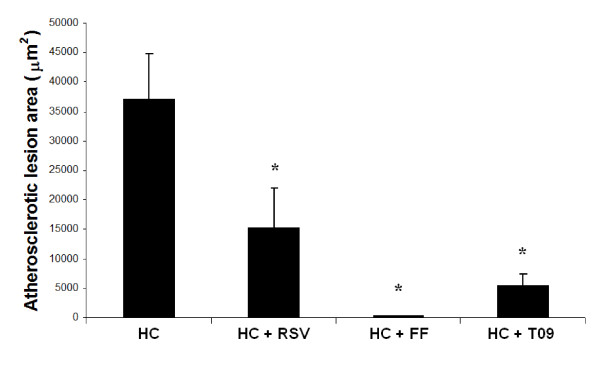
**Validation of *in vivo *endpoint effects of the drugs on early atherosclerosis**. Atherosclerotic lesion area determined in the aortic valve area of ApoE3Leiden mice treated with an atherogenic high cholesterol diet (HC) in the presence or absence of rosuvastatin (RSV), fenofibrate (FF) and LXR agonist T0901317 (T09). *P<0.05 versus HC (ANOVA and least significant difference post-hoc testing).

To identify the processes and pathways specifically suppressed by FF during atherogenesis, we mapped the unique DEGs (i.e. list of 148 genes for FF; Figure 8B; blue arrow) on biological process maps using enrichment analysis. Remarkably, FF but not RSV or T09 suppressed pathways and processes important for early atherogenesis. For example, FF suppressed CDR4-mediated cell adhesion, leukocyte chemotaxis and migration, leukocyte/lymphocyte activation, and lymphocyte proliferation, which were among the highest ranked processes (Table 2 and Additional file 3). Notably, early-stage disease processes (immune cell adhesion and recruitment, leukocyte proliferation) were identified independently by prediction analysis of possible targets and by analyses of experimental gene expression profiles. Since the modeling and experimental data indicated that FF would be more anti-atherogenic than RSV and T09 (due to its distinct quenching effect on immune and inflammatory processes associated with early atherosclerogensis), a greater impact of FF versus RSV and T09 would be expected on *initiation *of atherosclerotic lesions. Analysis of early atherosclerosis in the aortic valve area indeed showed that FF is a very potent quencher of lesion formation (Figure 9).

**Table 2:** Biochemical validation of the most significant pathways and biological processes unique for fenofibrate.

GeneGo Pathway Maps	pValue	GO Processes	pValue
Oxidative phosphorylation	7.737e-07	lymphocyte proliferation	2.476e-15
Chemotaxis: CCR4-induced leukocyte adhesion	3.564e-05	mononuclear cell proliferation	5.757e-15
Protein folding: Membrane trafficking and signal transduction of G-alpha (i) heterotrimeric G-protein	2.201e-04	leukocyte adhesion	2.324e-13
Neurophysiological process: Dopamine D2 receptor transactivation of PDGFR in CNS	5.723e-04	cell proliferation	3.118e-13
Immune response: Antigen presentation by MHC class I	6.410e-04	leukocyte migration during inflammatory response	6.116e-12
Transcription: Ligand-Dependent Transcription of Retinoid-Target genes	1.164e-03	endothelial cell migration	1.127e-10
Cell adhesion: Alpha-4 integrins in cell migration and adhesion	1.271e-03	lymphocyte activation	1.994e-10
Chemotaxis: Leukocyte chemotaxis	1.343e-03	leukocyte activation	3.843e-10
Immune response: Role of integrins in NK cells cytotoxicity	1.759e-03	activated T cell proliferation	6.308e-10
Signal transduction: cAMP signaling	1.759e-03	T cell proliferation	9.645e-10

Differentially expressed genes unique for fenofibrate (obtained from the microarray gene expression) were employed to GeneGo Pathway Maps and Gene Ontology (GO) Processes analysis. The 10 most significant pathways maps and biological processes are shown.

## Discussion

A systematic strategy that relies on both *in silico *modeling and experimental data was developed to help predict the origins of drug actions. Our systems biology strategy may also provide a rationale for explaining differential *and *common effects of drugs and drug classes. The prediction method uses information derived from the chemical structure of the drugs combined with experimental omics data obtained from short-term efficacy studies. Overall, we demonstrated notable consistencies between the computationally predicted effects and the wet-lab biochemical effects. The results suggested that the systems biology-based approach may facilitate identification of on-target and off-targets effects of a new drug which can be determined in the early stages of the drug development cycle. As importantly, the strategy may improve understanding of drug efficacies and aid in predicting safety leading to reduced costs of drug development and drug attrition rates. This approach may have important applications and implications for preclinical research and for development of novel therapeutic strategies including drug combinations.

Prototype cardiovascular drugs were used to evaluate our strategy. Differential effects on lipid metabolism and inflammation as obtained from *in silico *predictions were experimentally confirmed. A key step of the strategy is the use of computational predictions using structural data of the parent compound, compounds in the same chemical class, and their metabolites. The power of this approach is that variation in the structure of compounds belonging to a specific class allows a greater chance of finding other protein-protein interactions that are relevant for understanding primary and secondary effects of the drug. The MetaDrug database is very large and contains over 700,000 chemical structures and approximately 500,000 protein-compound interactions (covering about 4,500 protein targets), increasing the number of chemically-similar compounds and resulting in an increased power of the analyses. An added strength of this study was the ability to computationally expand from primary targets to their closest network interactions. Expanding the network increased the number of pathways and genes that could be compared thereby increasing the ability to predict effects on biological processes involved in the phenotype or disease process.

The hypotheses generated by these prediction tools and database were experimentally validated by (i) *in vitro *and *in vivo *techniques that quantified plasma levels of lipids and inflammation markers, (ii) by analyzing activity of key transcription factors in livers, and (iii) analysis of atherosclerotic plaque size in the aorta. It was predicted that the biological pathways maps that RSV, T09 and FF would affect would hardly overlap and that similarities between these drugs could mainly be expected within the maps of *lipid metabolism *and *inflammation*. Among the processes predicted to be affected by all three drugs were IL-1 signaling and MIF signaling and among the 21 common targets predicted were PPARγ, C/EBP, SMAD3, p53, SP1, Rb protein, COX-2, ERK1/2 and JNK. *In silico *network analyses also predicted that FF would affect the most network processes and that FF would differ in its impact on inflammation (with RSV and T09 being more similar). The experimental validation using the ApoE*3Leiden mice treated with the drugs specifically focused on inflammatory networks. The data confirmed the predictions: all three drugs affected inflammatory processes controlled by IL-1 and MIF and mediated by C/EBP, SP1, ERK1/2 and JNK. Also, the drugs showed a specific pattern of anti-inflammatory action with RSV and T09 sharing more similarities (for instance activation of central transcription factors C/EBPβ and p65-NFκB). In accordance with the model prediction, FF differed markedly from RSV and T09 in its overall effects on inflammation. While RSV and T09 mainly affected acute inflammatory response processes, FF was more effective in controlling chronic inflammation processes. A notable prediction was that FF would quench IL-6 signaling and related downstream effects relative to RSV and T09, which was confirmed at the protein level by transcription factor activity analysis (reduced STAT3 activity) and by the observed FF-specific reduction of circulating fibrinogen, an IL-6-inducible acute phase protein and chronic inflammation marker. Collectively, comparative genome-wide pathway mapping showed that the biological effects of the drugs were realized largely via different pathways and mechanisms suggesting complementarities.

Transcriptome data was used to predict physiological effects relevant for the vasculature and, to a lesser extent, for the liver itself. For example, FF (but not RSV or T09) was predicted to have an effect on processes important for leukocyte activation, migration and recruitment, all of which are crucial processes in early atherogenesis. Indeed, FF had the strongest effect on early atherosclerotic lesion development under the applied experimental conditions. Thus, while all three drugs were expected to be anti-atherosclerotic, FF is predicted to quench immune and inflammatory processes which play an important role in early lesion formation. This effect distinguishes FF from RSV and T09 and suggested that FF would be more potent than the other two drugs. Indeed, FF had the strongest reducing effect on early atherosclerosis. Extending the observations for processes and drug effects to other tissues will require additional confirmatory experiments. Although an increasing number of published studies use transcriptomic data from circulating cells to extrapolate to biological effects in other tissues and the effects of drugs [28], insufficient data exists to estimate the value and limitations of these strategies.

An increasing number of studies have shown that several cardiovascular drugs originally designed to lower plasma lipid levels also have beneficial anti-inflammatory effects, specifically the down-regulation of major inflammatory markers (TNFα, interleukin-1β, fibrinogen, SAA and CRP) and several key inflammatory transcriptional regulators (NF-κB). These effects were described by us and others for hypolipidemic drugs of different classes: statins, fibrates and LXR agonists [15,29-33]. However, the pathways and mechanisms that explain these anti-inflammatory effects remained largely unknown including whether these drugs act on the same or different pathways. Our data showed differential activities on inflammatory processes with signaling pathways and specifically via the key regulators including interferon-gamma, TGFβ, IL-1, TNFα, MIF and IL-6. These experimental data indicated that the profound inflammation quenching effect of FF may be through its effect on IL-6 signaling, a result consistent with the global suppression of IL-6-regulated genes by FF [34] and its negative (PPAR-alpha-dependent) effect on the IL-6 target gene fibrinogen [35]. However, the targets of FF and T09 are nuclear hormone receptors which are expressed in a cell- and tissue-specific manner and our observations are based on analyses of liver after chronic exposure to these drugs. Whether similar effects apply to other tissues and whether these affects also persist under conditions of chronic drug exposure remains to be experimentally tested. Many current systems biology-based strategies rely upon data from one organ that is composed of multiple types of cells, a distinct limitation of existing tissue isolation and analyses technologies.

Functional systems and pathway analyses methods capable of analyzing complex, multi-gene biological phenotypes are rapidly developing and are likely to help in understanding the mechanisms of drug effects. A structured "knowledge base" consisting of protein-protein interactions, pathways and processes assembled in ontologies [16] is required for such analyses. The data used to generate the lists of molecules (genes, proteins, metabolites) for prediction and pathway analysis may, however, be derived from experiments in different species, methods, and strategies. As shown here, the ability to correctly predict experimental results indicated the utility and potency of systems biology strategies in general and for translating results from laboratory animals models to the human. Nevertheless, these strategies are currently a method for hypothesis generation and the results, however promising, have to be considered "predictions." The three compounds analyzed are well known drugs which have a lot of associated publications in the literature and consequently in the MetaDrug database. The predictions made herein are, however, only partially based upon existing literature connections. On basis of the chemical structure of the compounds, our method also extrapolates to possible metabolites (formed after liver passage) and their respective targets - this portion of the prediction process is solely based on the chemical structure of the drugs and can be viewed as true predictions.

Developing new drugs is a tedious and expensive undertaking. Despite improvements in rational drug design and high throughput screening methods, the number of novel, single-target drugs fell greatly behind expectations during the past decade. In addition, the treatment of complex diseases involving multiple genes and risk factors remains a pressing medical need. The effects of drugs on known or unsuspected targets present both opportunities and challenges for modern drug discovery. Developing high-efficacy drugs that alter the activity of multiple targets or repositioning existing drugs to treat different diseases highlight the possibilities of a systems biology approach. However, off-target effects may result in adverse drug reactions that account for around one-third of drug failures during development and may contribute to idiosyncratic drug-induced damage to tissues. Reliable and reproducible strategies and models for predicting efficacy and safety, particularly in being able to identify the direct and indirect targets early in the drug development process are greatly needed. Such strategies are increasingly relevant for the development of successful combination therapies for patients suffering from complex, multifactorial cardiometabolic pathologies. Examples include patients treated with one or more drugs such as lipid-lowering and/or hypotensive drug therapies. This report provides an example of and extends the scope of systems biology approaches for drug discovery.

## Conclusions

We have developed a generic cheminformatic strategy that is applicable to any chemical entity and that allows the prediction of drug effects on biological processes. The method is based on a very large database containing 700,000 chemical structures and approximately 500,000 protein-compound interactions. The new method can provide mechanistic rationales for the differential and common effects of pharmaceuticals as demonstrated for three prototype cardiovascular drugs (a statin, a fibrate and an LXR activator). The biological pathway activity of these liver-targeting drugs was predicted using MetaDrug software and the results were tested against gene expression and protein measurements from a humanized mouse model of atherosclerosis. Consistent with the predictions, the drugs suppressed different facets of inflammation and displayed differential efficacy on early atherogenic processes and cardiovascular endpoints (atherosclerotic lesion area). This study exemplifes the power of computational strategies for efficacy prediction and the role that systems biology may have in future drug discovery.

## Methods

### Animal atherosclerosis experiment

12-week old female ApoE*3Leiden transgenic (E3L) mice were used. E3L mice express a 27 kb genomic region from an ApoE*3Leiden proband encoding the human APOE*3Leiden gene, the APOC1 gene, and all their known regulatory elements, including those for liver-specific expression [9]. Animal experiments were approved by an Independent Animal Care and Use Committee (DEC) and were in compliance with European Community specifications regarding the use of laboratory animals.

All animals were fed an atherogenic diet consisting of (all w/w) 20% casein, 40.5% sucrose, 15% cocoa butter, 1% corn oil, 10% corn starch, 0.25% standard vitamin, 0.25% mineral premix, 0.7% CaHPO_4_, 1% CaCO_3 _0.7% KH_2_PO_4_, 0.7% KCl, 0.3% NaCl, 0.4% MgSO_4_, 0.2% MgO, 0.2% methionine, 2% choline CL, 6.2% dicacel2+4 cellulose (Hope Farms, Woerden, The Netherlands). This diet was fed for 10 weeks to a reference control group (Con group). A high cholesterol group (HC group) received the same diet but supplemented with 1% (w/w) cholesterol to induce atherosclerosis. Atherosclerosis and transcriptomics data of these two groups were published separately[27]. All drug treatment groups were fed the same high cholesterol diet as the HC group but supplemented with one of the following: PPAR-alpha activator fenofibrate (FF; 0.03% w/w), HMG CoA-reductase inhibitor rosuvastatin (RSV; 0.05% w/w), or LXR-activator, T-0901317 (T09; 0.01% w/w). After 10 weeks of diet feeding, animals were euthanized to collect livers and hearts (including the aortic root). Livers were snap-frozen in liquid nitrogen and stored at -80°C until use for transcriptomics and metabolomics analysis. Hearts were fixed in formaldehyde for analysis of atherosclerotic lesions [36]. Cross sections were analyzed blindly in four cross-sections of each specimen (at intervals of 30 μm) [14]. Significance of difference in atherosclerosis lesion area was calculated by 1-way analysis of variance (ANOVA) test followed by a least significant difference post hoc analysis using SPSS 11.5 for Windows (SPSS, Chicago, USA). The level of statistical significance was set at α<0.05.

### Analysis of plasma lipids and proteins

Total plasma cholesterol and triglycerides were measured using kits No.1489437 (Roche Diagnostics, Almere, The Netherlands) and No.337-B (Sigma, Aldrich Chemie BV, Zwijndrecht, The Netherlands) [36]. The levels of the liver-derived inflammation markers serum amyloid A (SAA) and fibrinogen were determined by established ELISAs [36].

### Nucleic acid extraction and microarray analysis

Nuclear acid extraction and gene expression data analysis have been performed as described previously in detail [27]. Briefly, total RNA was extracted (n = 5 livers per group) using glass beads and RNAzol (Campro Scientific, Veenendaal, The Netherlands). Integrity of obtained RNA was examined using the RNA 6000 Nano Lab-on-a-Chip kit and a bioanalyzer 2100 (both Agilent Technologies, Amstelveen, The Netherlands). Biotinylated cRNA (from 5 μg of total RNA) was prepared with a One-Cycle Target Labeling and Control Reagent kit (Affymetrix #900493). Intermediate products, i.e. biotin-labeled cRNA and fragmented cRNA, were again checked (Agilent bioanalyzer). Microarray analysis was carried out using Affymetrix mouse GeneChip^® ^430 2.0 arrays containing 45,037 probe sets and 34,000 well-characterized mouse genes. Fragmented cRNA was mixed with spiked controls, applied to Affymetrix Test chips, and good quality samples were then used to hybridize with arrays. The hybridization, probe array washing and staining procedures were executed as specified by Affymetrix, and probe arrays were scanned with a Hewlett-Packard Gene Array Scanner.

### Gene expression data analysis

Raw signal intensities were normalized using the GCRMA algorithm (Affylm package in R). Datasets are freely accessible online through ArrayExpress database http://www.ebi.ac.uk/arrayexpress. The datasets of the control groups and drug treatment groups can be accessed at ArrayExpress under E-TABM-253 and E-MEXP-3282, respectively. Normalized signal intensities below 10 were replaced by the value of 10. Probe sets with an absent call in all arrays were removed before further analysis of the data. The gene expression changes obtained by microarray were positively validated in a head-to-head comparison by RT-PCR for the genes HMG-CoA reductase, lipoprotein lipase, apolipoprotein A1, serum Amyloid A1, fibrinogen, ATP transporter A1 and ATP transporter G1 in a previous study [27].

Statistical analysis was performed in BRB ArrayTools (Dr. Richard Simon and Amy Peng Lam, http://linus.nci.nih.gov/BRB-ArrayTools.html). Groups were tested for differentially expressed genes using class comparisons with multiple testing corrections by estimation of false discovery rates (FDR). Differentially expressed genes were identified at a threshold for significance of α<0.01 and a FDR<5%. Within the set of differentially expressed genes, a Student's *t*-test was carried out to test whether individual genes were differentially expressed between two groups (P<0.01 was considered significant).

### Prediction of biological effects of small molecule compounds using MetaDrug™

A detailed methodological description of the systems biology procedures and protocols to study of drug effects with details on experimental design, omics data handling and a step-by-step protocol for using MetaDrug™ (GeneGo, Inc.) software has been published [37]. Briefly, in a two-step workflow, the chemoinformatics tools in MetaDrug™ transform the chemical structure of a compound of interest into a list of potentially affected proteins allowing the prediction of biological effects (Figure 1). In the first step, human metabolites were predicted for the uploaded structure for RSV, FF and LXR by a set of empirical metabolic rules. The resulting list of metabolites and the parent compounds were then queried by a chemical similarity search against a MetaDrug™ database of some 700,000 manually annotated compounds linked via physical compound-protein interactions with some 4,500 proteins known as targets for at least one small molecule xenobiotic. Accord Chemistry Cartridge™ (Accelrys, San Diego, USA) fragment based fingerprints were applied to perform the similarity search.

This generated a list of compounds similar to the structure with a chosen similarity score (Tanimoto coefficient). The list of similar compounds retrieved the list of their protein targets using the collection of protein-target interactions annotated and stored in the MetaDrug™ database. In a next step, the protein target lists for RSV, FF and T09 were computationally expanded by their nearest neighbors, i.e. by their first-step physical protein-protein interaction partners, and intersected to identify common (shared by all compounds), similar (shared by any two compounds) and unique targets for every compound. Common and unique lists were subjected to enrichment analysis. Methodological and practical details about the use of the Compare Experiment Workflow, Gene List Enrichment Analysis, Network Generation, Transcription Factor Analysis and Network Expansion are described in a recent methodological paper [37]. The current limitations of the tools available for pathway mapping to the study of cardiovascular disease have been summarized in [38].

### Ontology enrichment analysis

We identified and ranked cellular pathways and processes most influenced by the uploaded compounds by enrichment analysis (EA) in two GeneGo ontologies (GeneGo Pathway Maps, GeneGo Toxicity Networks) and in GO Processes. Every map or network in a given ontological category consists of proteins or endogenous compounds (e.g. secondary messengers) participating in a pathway or a process (GeneGo Pathway Maps, GO Processes) or implicated in a disease or toxic state (GeneGo Toxicity Networks) linked by well-established interactions. The significance of enrichment was defined by p-values of hypergeometric distribution [16]. As a result, each compound was associated with a quantitatively ranked list of processes and diseases summarizing its pharmacological and toxic effects at a systems-biology level.

### Analysis of activated pathways and transcription factor binding activity

STAT3-DNA, AP-1 (c-jun), C/EBP-DNA and p65-NFkB binding activity was assessed using ELISA-based transcription factor assay kits #45196, #46096, #44196 and #40097-Chemi (TransAM™; Active Motif, Rixensart, Belgium) following the protocols provided by the manufacturer. Briefly, homogenous liver extracts were prepared using 'Active Motif Nuclear Extract Kit' and analyzed in 96-well microplates coated with the oligonucleotide-containing consensus binding site for the respective transcription factor. Thereafter, a primary antibody against the transcription factor was added, followed by the addition of a horseradish peroxidase-conjugated secondary antibody. Colorimetry was performed with tetramethylbenzidine, and optical density was read using a spectrophotometer at 450 nm with a reference wavelength of 650 nm. Specific for p65-NFkB, activity was determined by chemiluminescence using a luminescent image workstation (Roche Diagnostics, Almere, The Netherlands). The active phosphorylated form of IkB-α was quantified by a Functional IkB-α ELISA™ (Active Motif).

## Authors' contributions

RK and TK designed the experiment. RK, SB LV, AP and TK interpreted the data. RK, SB and TK wrote the main paper. LV performed the experiments and analyzed atherosclerosis. YN developed the concepts for the chemoinformatics approach. AP, SB conducted the bioinformatical analysis and, together with YN, interpreted the data. JK, EHC, BvO, YN, PW and AP edited the manuscript and gave conceptual advice. All authors read and approved the final manuscript.

## Authors information (Conflict of interest)

R.K., T.K., E.H.C., P.W., B.v.O. and J.K. do not have conflict of interest. S.B., A.P. and Y.N. are employees of GeneGo Inc. GeneGo Inc., a provider of data mining and analysis solutions in systems biology such as the MetaDrug software used for the present study.

None of the authors will however receive additional salary, additional personal income, or any form of financial support.

The views presented in this paper do not necessarily represent or reflect those of the U.S. FDA.

## Supplementary Material

Additional file 1 **Differential *in vivo *effects of cardiovascular drugs on plasma lipids**. Click here for file

Additional file 2 **Lists of potential targets obtained from the hepatic gene expression profile and lists of putative targets predicted from the chemical structure**. Click here for file

Additional file 3 **Differential effect of cardiovascular drugs on immune cell recruitment/chemotaxis**. Click here for file
