# Supplementary material for: A Systems Biology Strategy for Predicting Similarities and Differences of Drug Effects: Evidence for Drug-specific Modulation of Inflammation in Atherosclerosis
Source: BMC Syst Biol. 2011 Aug 12;5:125. doi: 10.1186/1752-0509-5-125 (PMC3163556; doi:10.1186/1752-0509-5-125)
Supplement: Additional file 1 — Differential in vivo effects of cardiovascular drugs on plasma lipids. [file 1752-0509-5-125-S1.DOC]

**Additional file 1**

**Differential *in vivo* effects of cardiovascular drugs on plasma lipids.**

This supplementary information summarizes the effects of the three drugs on plasma lipids. ApoE3Leiden transgenic mice were fed an atherogenic, 1% w/w cholesterol diet containing rosuvastatin (RSV), fenofibrate (FF), LXR activator (T09) or placebo (HC group) for 10 weeks. A reference control group (Con group) received the same atherogenic diet without cholesterol supplement. The average starting body weight for animals was 20±1 g. Body weight increased in all groups comparably and insignificantly (by 1 to 2 grams), except in T09 group which remained at the initial weight (Table S1). Food intake was comparable between the groups (2.5 to 2.6 g/d per mouse), except for the T09 group which consumed less (2.2 g/d; P<0.05).

**Table S1: Clinical chemistry of ApoE3Leiden mice fed an atherogenic diet and treated with cardiovascular drugs.** The average starting body weight was 20±1 g and the body weight at endpoint (week 10) is listed. The plasma levels of cholesterol and triglycerides were determined in fasting plasma over time and endpoint concentrations are provided. Data represent mean ± SD.*P<0.05 indicates significant difference compared to HC.

|  | **Con** | **HC** | **RSV** | **FF** | **T09** |
| --- | --- | --- | --- | --- | --- |
| **Body weight (g)** | 21±2 | 21±1 | 21±1 | 22±1 | 20±1 |
| **Average food intake  (g/d per mouse)** | 2.6±0.2 | 2.5±0.2 | 2.5±0.2 | 2.6±0.1 | 2.2±0.2* |
| **Cholesterol (mM)** | 6±1* | 19±4 | 14±3* | 8±1* | 23±4* |
| **Triglyceride (mM)** | 1.5±0.2* | 1.8±0.4 | 1.5±0.3* | 0.5±0.1* | 12.3±4.4* |

Plasma cholesterol in the Con group receiving cholesterol-free control diet was 6 mM. Feeding with a HC diet resulted in a stable and high plasma cholesterol level (19 mM in week 10 in the HC group) (Table S1). Treatment with RSV or FF reduced plasma cholesterol levels by 26% and 58%, respectively, while treatment with T09 had an opposite effect (plasma cholesterol concentrations elevated by 21%). Plasma triglyceride levels with the HC diet were 1.8 mM and RSV and FF had a significant triglyceride-lowering effect (17% and 72%, respectively), while T09 strongly and significantly elevated (680%) plasma triglycerides (Table S1).
