# Supplementary material for: A Systems Biology Strategy for Predicting Similarities and Differences of Drug Effects: Evidence for Drug-specific Modulation of Inflammation in Atherosclerosis
Source: BMC Syst Biol. 2011 Aug 12;5:125. doi: 10.1186/1752-0509-5-125 (PMC3163556; doi:10.1186/1752-0509-5-125)
Supplement: Additional file 3 — Differential effect of cardiovascular drugs on immune cell recruitment/chemotaxis. [file 1752-0509-5-125-S3.DOC]

**Additional file 3**

**Differential effect of cardiovascular drugs on immune cell recruitment/chemotaxis.** Many genes on the map were down-regulated by FF (thermometer number 2) but up-regulated by RSV and T09 (thermometers 1 and 3, respectively) or changed by RSV and T09 only.

| 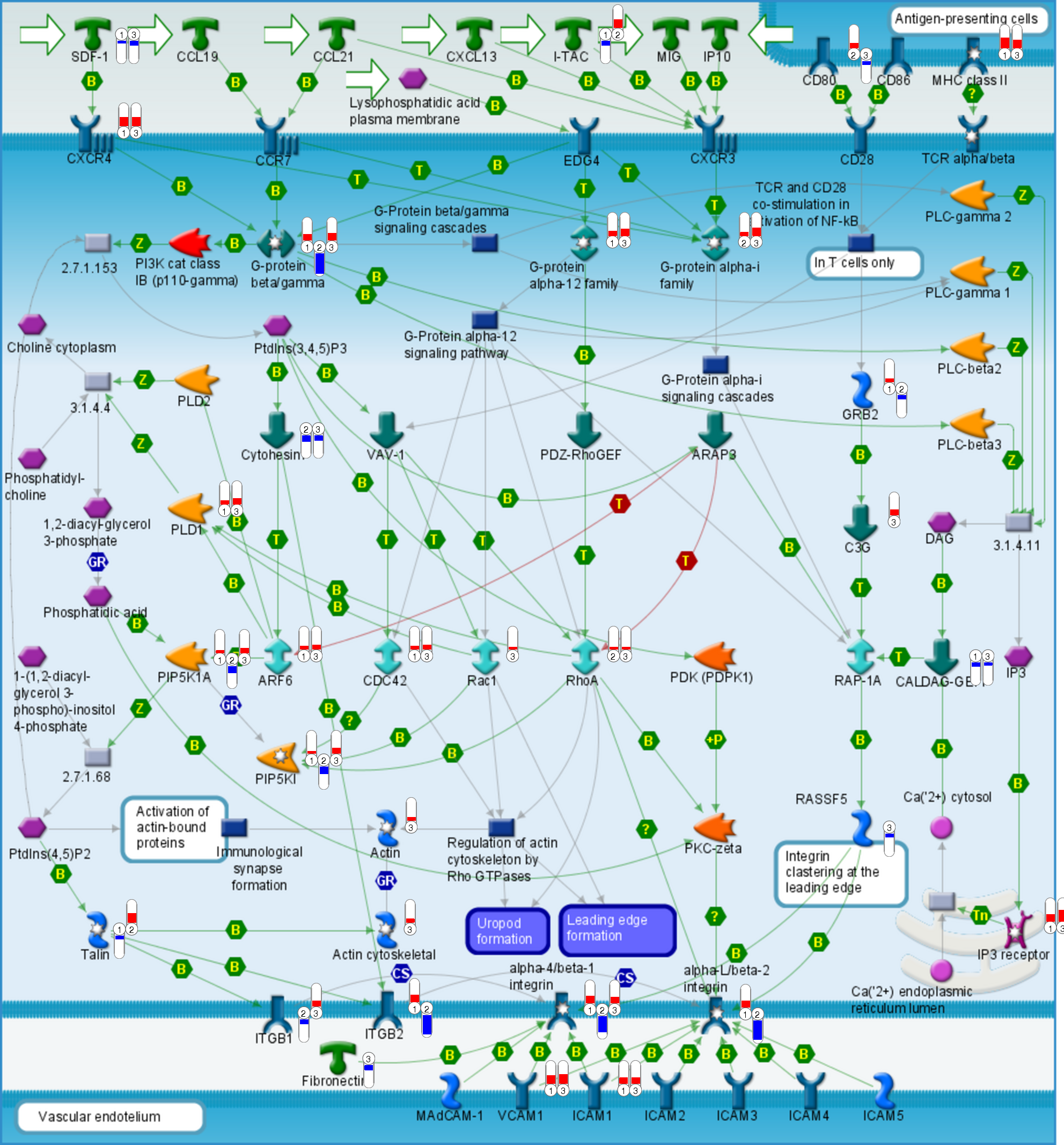 |
| --- |
| **Figure S1.** Unique genes down-regulated by fenofibrate belonging to the biological process map ‘Leukocyte Chemotaxis’. Proteins expressed differentially with rosuvastatin and T09 treatment compared with fenofibrate are highlighted by red solid rectangles. Proteins expressed by rosuvastatin and T09 only are highlighted by red dashed rectangles. Thermometer numbers indicate: 1– RSV, 2 – FF, 3 – T09. |

For instance, vascular cell adhesion molecule-1 (VCAM-1), intercellular adhesion molecule-1 (ICAM-1) and integrins are downregulated by FF but up-regulated by RSV and T09. These genes code for proteins that are important mediators of atherosclerosis plaque formation and induce firm adhesion of inflammatory cells. The differences in expression of adhesion genes and leukocyte proliferation between FF and RSV and T09 may be the key to the different efficacy of the drugs in early atherogenesis. The two processes were identified independently by enrichment analysis of the predicted targets and the expression profiles which confirms the significance of the processes in the FF mode of action.
